# Technology-enhanced simulation-based learning in orthodontic education: A scoping review

**DOI:** 10.1590/2177-6709.28.3.e2321354.oar

**Published:** 2023-07-17

**Authors:** Kawin SIPIYARUK, Prachworrakit KAEWSIRIRAT, Peerapong SANTIWONG

**Affiliations:** 1 Mahidol University, Faculty of Dentistry, Department of Orthodontics (Bangkok, Thailand).

**Keywords:** Dental education, Orthodontics, Serious game, Simulation, Technology-enhanced learning

## Abstract

**Introduction:**

Technology-enhanced simulations seem to be effective in dentistry, as they can support dental students to improve competencies in simulated environments. However, implementation of this technology in orthodontic education has not been reviewed.

**Objective:**

This scoping review aimed to comprehensively summarize the use of technology-enhanced simulations in orthodontic practice.

**Methods:**

A systematic search was conducted to identify literature on technology-enhanced simulation-based learning in orthodontic education published from 2000 to 2021. The search was conducted up to September 2021 to identify articles from Scopus, Embase, PubMed, ProQuest Dissertations & Theses Global, Google Scholar and the reference lists of identified articles.

**Results:**

The search identified 177 articles. Following the inclusion and exclusion criteria, 16 articles of 14 digital simulators were included in this review. The findings demonstrated an increasing use of technology-enhanced simulations in orthodontic education. They were designed in several formats, including three-dimensional virtual format, augmented reality, virtual reality, automaton, haptic, and scenario-based simulations. These simulations were implemented in varied areas of orthodontics including diagnosis and treatment planning, bracket positioning, orthodontic procedures, facial landmark, removable appliance and cephalometric tracing. Most included articles demonstrated the development process without outcome evaluation. Six studies provided outcome evaluations at reaction or learning levels. None of them provide the evaluation at behaviour and results levels.

**Conclusion:**

Insufficient evidence has been generated to demonstrate the effectiveness of technology-enhanced simulations in orthodontic education. However, high-fidelity computer-based simulations together with robust design research should be required to confirm educational impact in orthodontic education.

## RESUMO

**Introdução:**

Simulações aprimoradas por tecnologia parecem ser eficazes em Odontologia, pois podem ajudar os estudantes a melhorar suas competências em ambientes simulados. No entanto, a implementação dessa tecnologia na educação ortodôntica ainda não foi revisada.

**Objetivo:**

A presente revisão de escopo teve como objetivo resumir, de forma abrangente, o uso de simulações aprimoradas por tecnologia na prática ortodôntica.

**Métodos:**

Uma busca sistemática foi realizada para identificar publicações de 2000 a 2021 sobre aprendizado na educação ortodôntica baseado em simulação aprimorada por tecnologia. A busca foi realizada até setembro de 2021 para identificar artigos na Scopus, Embase, PubMed, ProQuest Dissertations & Theses Global, Google Scholar e nas listas de referências dos artigos identificados.

**Resultados:**

A busca identificou 177 artigos. Foram incluídos nessa revisão 16 artigos, com 14 simulações digitais, que atenderam aos critérios de inclusão e exclusão. Os resultados demonstraram um uso crescente de simulações aprimoradas por tecnologia na educação ortodôntica. Elas foram projetadas em vários formatos, incluindo formato tridimensional virtual, realidade aumentada, realidade virtual, autômato, háptico e simulações baseadas em cenários. Essas simulações foram implementadas em diversas áreas da Ortodontia, incluindo diagnóstico e planejamento de tratamento, posicionamento de braquetes, procedimentos ortodônticos, identificação de pontos de referência faciais, aparelhos removíveis e traçados cefalométricos. A maioria dos artigos incluídos demonstrou o processo de desenvolvimento, sem avaliar os resultados. Seis estudos forneceram avaliações de resultados em níveis de reação ou aprendizado. Nenhum deles forneceu a avaliação em níveis de comportamento e resultados.

**Conclusão:**

Não foram geradas evidências suficientes para demonstrar a eficácia das simulações aprimoradas por tecnologia na educação ortodôntica. No entanto, simulações de alta fidelidade baseadas em computador, juntamente com pesquisas robustas de design, são necessárias para confirmar o impacto educacional na área ortodôntica.

## INTRODUCTION

The COVID-19 pandemic has widely impacted a variety of areas, including the educational field. In this context, emphasis on hybrid learning (presential/distance) is unavoidable to minimize the risk of infection. The concerns of physical distancing have been raised in healthcare education, including orthodontic practice. Unlike other educational areas, the emphasis of orthodontic education is to improve psychomotor skills, in addition to cognitive and affective domains.^1^ Technology-enhanced simulation-based learning can be designed in varied formats, such as digital simulators, augmented reality (AR), virtual reality (VR) and serious games, to enhance knowledge and skills in dental practice.^2^ ^-^ ^4^ All of these options should be considered for orthodontic training. 

Orthodontic practice requires knowledge and skills in various areas, such as anatomy of head and neck, growth and development, physiology and biomechanics of tooth movement.^5^ ^,^ ^6^ Furthermore, the learning outcomes of orthodontic programs generally require varied psychomotor skills, such as wire bending, bracket positioning, and tooth stripping for interproximal reduction.^6^ Residents are also required to be competent in the affective domain, to communicate with patients and their guardians or to deal with orthodontics-related psychological concerns among patients.^7^ Therefore, learning and practice in laboratories followed by clinical settings should be necessarily designed for all orthodontic postgraduate programs. 

The COVID-19 outbreak seems to have negative impact on orthodontic practice, evidenced by a delay of orthodontic treatment caused by the lockdown or quarantine.^8^ ^,^ ^9^ There is evidence demonstrating a decrease in the number of new-patient visits during the pandemic.^10^ Patients may feel unsafe or experience restrictions to attend the orthodontic appointments.^11^ ^,^ ^12^ They may also have financial problems related to the pandemic.^12^ A survey in 69 dental schools found a restriction of clinical practice during the pandemic, as only urgent or emergency services were permitted.^13^ This situation seems to be a major challenge for orthodontic education, where training in clinical settings is highly required.^14^ The consideration of appropriate substitutes to clinical practice should be required to ensure that graduates will be able to achieve the expected learning goals. 

Unlike a cognitive domain, which can be replaced by an online format or a hybrid learning model, orthodontic practice requires clinical techniques learning, where technology-enhanced simulation-based learning can play an important role to offer substitutes or supplements to clinical practice. There is evidence reporting that digital simulators can enhance psychomotor skills, preparing dental undergraduates for practice in clinical settings.^15^ ^,^ ^16^ These technology-enhanced simulations can be applied in various fields of dental education, including prosthodontics, endodontics, maxillofacial surgery, periodontology, radiology, pediatric dentistry and orthodontics.^17^

Despite the high setup costs and trained staff required, technology-enhanced simulators can be considered more effective than traditional simulations, in terms of unlimited training sessions with objective and repetitive feedback,^2^ resulting in higher efficiency of teaching and learning in dental education. 

Although technology-enhanced simulation-based learning has been used to assist dental education, no review of its implementation into clinical training of orthodontic practice could be found. Consequently, this scoping review was conducted to comprehensively analyze empirical studies of the use of technology-enhanced simulation-based learning in orthodontic practice. The knowledge and understanding retrieved from this review would be supportive for dental educators to systematically and comprehensively consider the design and implementation of technology-enhanced simulations to provide optimum settings for teaching and training in orthodontic education. 

## METHODS

### REVIEW DESIGN

A scoping review of the literature was considered as the most appropriate method to synthesize the use of technology-enhanced simulation-based learning. The purposes of this type of review are to identify key concepts, characteristics, available evidence and research gaps of an interesting topic.^18^ This design is also appropriate for a complex issue, especially when it has not been yet comprehensively reviewed.^19^ The review process comprises six stages, as follows: 1) identify research questions or purposes, 2) identify relevant literature through systematic searches, 3) select articles in accordance with inclusion and exclusion criteria, 4) analyze the data retrieved from the identified evidence, 5) collate, summarize, and report results; and 6) consult external stakeholders for further suggestions or insights to the review (optional).^20^ This report follows the PRISMAS checklist for scoping reviews.^21^

### RESEARCH QUESTIONS

This scoping review sought to answer the following questions:

» What was the trend in the current use of technology-enhanced simulation-based learning in orthodontic education?
» What types of technology-enhanced simulation-based learning were made available to orthodontic education?
» What were educational outcomes of available technology-enhanced simulation-based learning in orthodontic training?

### SEARCH STRATEGY

Literature search was performed across four databases, including Scopus, Embase, PubMed, and ProQuest Dissertations & Theses Global. Google Scholar and the reference lists of identified articles were also screened for relevant literature. Search terms was developed following the PICOS strategy,^22^ including: Population = ‘Orthodontic student’ and ‘Orthodontist’; Intervention = ‘Simulation’, ‘Virtual reality’, ‘Augmented reality’, and ‘Video game’; Comparison = ‘No intervention’ and ‘Traditional approach’; Outcome = ‘Knowledge’, ‘Skill’, and ‘Competency’; and Study = ‘Any type of studies’. However, only ‘Population’, ‘Intervention’, and ‘Outcomes’ were implemented, as well as ‘orthodontic’ and ‘orthodontics’ were used instead of ‘Orthodontic student’ and ‘Orthodontist’, to extend the search, covering as many as available publications. As several articles identified from the initial search were technical reports demonstrating only the development process of simulations, the terms ‘training’, ‘education’, ‘learning’, and ‘teaching’ were considered for ‘Outcome’, rather than ‘Knowledge’, ‘Skill’, and ‘Competency’. Moreover, the search terms for ‘Outcome’ were still required to enhance the emphasis on the use of technology-enhanced simulation on educational purposes, rather than its use as a process of orthodontic treatment. The search process was iteratively performed and adjusted to ensure its robustness before conducting the final search.^23^ The last search was conducted on September 30, 2021.

### INCLUSION AND EXCLUSION CRITERIA

All types of empirical studies of technology-enhanced simulation-based learning in orthodontic education published from January 2000 to September 2021 were included in this review. Grey literature was also expected to cover technology-enhanced simulation in orthodontic education wherever possible; however, the references were excluded if fail to include technology-enhanced simulations or were not used for teaching or training orthodontic professionals or residents. They were also not included if not available in full-text. 

### STUDY SELECTION AND DATA EXTRACTION

All identified articles were screened by two researchers (KS and PK) to consider whether or not they were eligible for this review. Any disagreement on the decision was resolved by discussion with the other researcher (PS). The table of data extraction was developed following an iterative testing in extracting information from the included articles, with the discussion among researchers based on the research questions and literature review. The data were extracted covering authors, year of publications, learning topics, types and concepts of simulations, research objectives, study design and data collection methods, as well as reported educational outcomes of the simulations (Table 1). The data from included articles were extracted by a researcher experienced with systematic reviews (KS). The data extraction was then reviewed by another researcher (PS) to confirm the validity. Disagreement was settled by discussion among researchers (KS, PK, and PS) to achieve a consensus. 

**Table 1:** Information extracted from the included articles.

Authors (year)	Learning topics of simulations	Types and concepts of simulations	Research objectives	Educational outcomes of the simulations reported in research
Rodrigues et al. (2006)^33^	Orthodontic treatment planning and tooth movement	3D interactive simulation that allows users to manage with orthodontic problems and suggest possible treatments	To demonstrate the development of the prototype of 3D simulation for orthodontic treatment	Technical report without a data collection process and evidence of educational outcomes
Rodrigues et al. (2007)^24^	Orthodontic treatment planning and tooth movement	3D interactive simulation that allows users to gain experiences in treatment planning and tooth movement in simulated orthodontic patients, together with a treatment outcome prediction.	To demonstrate the development and validation process of the intervention	Technical report without a data collection process and evidence of educational outcomes
Rodrigues et al. (2008)^34^	Orthodontic treatment planning and tooth movement	3D interactive simulation that allows users to gain experiences in treatment planning and tooth movement in virtual patients	To present the implementation of discrete and continuous collision detection algorithms in the development of the intervention	Technical report without a data collection process and evidence of educational outcomes
Sinthanayothin and Tharanont (2008)^35^	Orthodontic treatment planning	3D computer-based simulation that allows users to gain experience of treatment plan in orthodontic practice	To demonstrate the development of 3D orthodontic treatment simulation	Technical report without a data collection process and evidence of educational outcomes
Yaqi and Zhongke (2010)^36^	Orthodontic treatment planning	3D computer-based simulation that allows users to gain experience of treatment plan in orthodontic practice	To demonstrate the development of 3D orthodontic treatment simulation	Technical report without a data collection process and evidence of educational outcomes
Kumar (2012)^39^	Orthodontic treatment planning	3D computer-based simulation that can support students to simulate and observe different treatment outcomes from different treatment plans	To demonstrate the development automation process of the simulation	No report of a data collection process and research outcomes in terms of educational and training purposes
Naser-ud-Din (2014)^25^	Clinical cases and procedures in orthodontic practice	Scenario-based learning interactive software that allows users to experience four orthodontic clinical cases and five orthodontic procedures, together with questions with immediate feedback provided	To evaluate the intervention using a questionnaire survey	The survey using an open-ended paper-based questionnaire demonstrated high acceptance level for the intervention, which could provide confidence in the application of clinical skills
Rao et al. (2017)^37^	Orthodontic bracket positioning	Augmented reality with haptic technology that allows users to gain cognitive and psychomotor skills, as well as confidence in bracket positioning of orthodontic practice	To propose the intervention, as well as its expected benefits	Technical report without a data collection process and evidence of educational outcomes
Rao et al. (2018)^38^	Cephalometric tracing in orthodontics	Augmented reality that allows users to learn various skeletal and soft tissue landmark points	To propose the intervention, as well as its expected benefits	Technical report without a data collection process and evidence of educational outcomes
Rao et al. (2019)^26^	Facial landmark points	Automation of facial landmark identification that allows students to perform multiple measurements of orthodontic landmark points on 2-D patient images as a part of orthodontic training	To evaluate the accuracy of algorithm in identifying facial landmarks on 2-D facial images	The study reported the benefit of the intervention as a valuable training tool in supporting learners to perform orthodontic facial analysis, by comparing it with a direct physical measurement with a caliper
Sakowitz et al. (2020)^27^	Diagnosis and treatment planning of orthognathic surgery	Virtual reality with Oculus VR headset that allows users to interact with a virtual patient using keyboard and mouse	To compare effectiveness of the virtual reality and a conventional 2D approach, by evaluating student understanding in diagnosis and treatment planning of orthognathic cases	The randomized controlled trial (with pre-, post-, and follow-up tests) demonstrated the improvements of understanding in diagnosis and treatment planning in both VR and conventional approaches, without significant difference between the two groups
Gredes et al. (2021)^28^	Removable orthodontic appliance, called AR-Demonstrator-App	Augmented reality that allows users to learn manufacturing steps of removable orthodontic appliance on a plaster model	To explore student perceptions on handling, acceptance, and usefulness of the intervention use	The survey using a paper-based questionnaire demonstrated the students tended to have positive attitudes toward the intervention
Ho et al. (2021)^29^	Orthodontic diagnosis	3D digital dental model with inquiry-based learning that allows undergraduate dental students to learn orthodontic diagnostics in simulated environment	To explore user perceptions toward the intervention and to investigate learning performance of students	The mixed-methods research (using a questionnaire and focus group discussion) demonstrated high acceptance of the intervention among dental students
Lo et al. (2021)^30^	Orthodontic bracket positioning	Augmented reality that supports orthodontists in bracket placement for orthodontic treatment (an assisted bracket navigation system)	To evaluate the accuracy of bracket placement by novice and expert orthodontists between AR and a conventional method	The clinical trial reported that the intervention could enhance the accuracy of bracket placement for novice orthodontists, by comparing the accuracy of bracket placement between the conventional and AR methods by measuring the deviations of bracket positions
Sytek et al. (2021)^31^	Diagnosis and treatment planning of orthognathic surgery	Virtual reality with Oculus VR headset that allows users to use touch controllers to interact with virtual patients in an immersive environment	To assess performance and attitudes for treatment planning in orthognathic surgery cases using 2D, 3D, and VR simulations among orthodontic residents	The mixed-methods research reported no significant difference in overall performance of residents among 2D, 3D, and VR simulations. In addition, the evidence from semi-structured interview demonstrated that the residents also tended to be ready to adopt VR simulation
Ye et al. (2021)^32^	Orthodontic bracket positioning	Haptic training simulation that allows users to perform the required steps of orthodontic bracket placement on a virtual patient	To demonstrate the design and development of the simulation, including the graphics engine	Technical report without a data collection process and evidence of educational outcomes

## RESULTS

### LITERATURE IDENTIFIED FROM THE SEARCH

**Figure 1:**
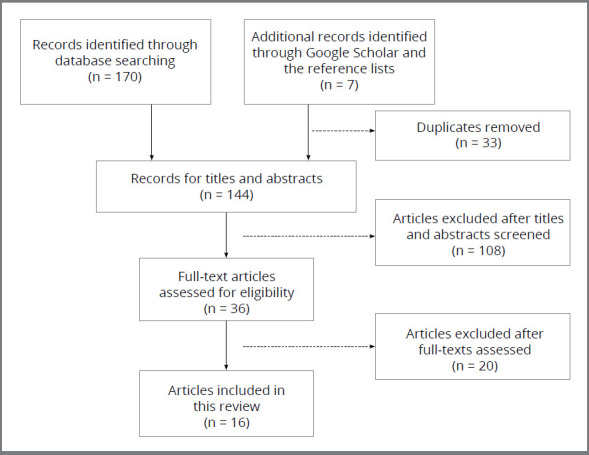
A flow diagram presenting the articles selection process for this review.

The search conducted across the four databases identified 170 articles. Google Scholar and the reference lists of identified articles were also screened, and seven papers were further identified. After that, 33 duplicates were removed and 144 titles and abstracts were reviewed. One hundred and eight articles were excluded, as they were reviews and/or not relevant to technology-enhanced simulations. Thirty-six full-texts were then assessed, and twenty articles were excluded: ten were technology-enhanced simulations used for only orthodontic treatment, rather than for training purposes; three were interventions that were not considered as technology-enhanced simulations; three were not related to orthodontics; two were reviews; one was a traditional simulation; and one was not available in full-text. The article selection process is presented in Figure 1. 

### CHARACTERISTICS OF INCLUDED ARTICLES

The sixteen articles included in this scoping review comprised nine journal articles,^24^ ^-^ ^32^ six conference papers,^33^ ^-^ ^38^ and one PhD thesis.^39^ Two articles were experimental designs comparing intervention and conventional approaches,^27^ ^,^ ^30^ and one of them was a randomized control trial.^27^ Two studies used only a questionnaire survey design to gather user perception toward the use of technology-enhanced simulations.^25^ ^,^ ^28^ Two articles reported the used of mixed methods design, where focus group discussion and semi-structured interview were used to collect qualitative data.^29^ ^,^ ^31^ Nine articles and one thesis demonstrated the development and validation process of computer-based simulations, with no report of data collection process.^24^ ^,^ ^26^ ^,^ ^32^ ^-^ ^39^ When considering the year of publication, over the 15-year period (2006 to 2020), only 11 publications were found introducing simulations (9 approaches),^24^ ^-^ ^27^ ^,^ ^33^ ^-^ ^39^ which nearly a half of them were design to facilitate orthodontic practice as a main purpose, although not applicable for training. Moreover, five articles had already been made available within the 10-month period in 2021.^28^ ^-^ ^32^

### CHARACTERISTICS OF TECHNOLOGY-ENHANCED SIMULATIONS INCLUDED IN THIS REVIEW

Of those 16 identified articles, 14 technology-enhanced simulations were introduced. Five simulations were designed in a three-dimensional (3D) format, as reported in seven articles;^24^ ^,^ ^29^ ^,^ ^33^ ^-^ ^36^ ^,^ ^39^ four simulations adopted an AR technology;^28^ ^,^ ^30^ ^,^ ^37^ ^,^ ^38^ two employed a VR technology to support immersive learning;^27^ ^,^ ^31^ one was scenario-based simulation;^25^ one was automation of facial landmark identification;^26^ and one reported the application of haptic technology.^32^ The identified computer-based simulations were implemented in varied topics in orthodontic education, covering diagnosis and treatment planning,^24^ ^,^ ^27^ ^,^ ^29^ ^,^ ^31^ ^,^ ^33^ ^-^ ^36^ ^,^ ^39^ orthodontic bracket positioning,^30^ ^,^ ^32^ ^,^ ^37^ orthodontic cases and procedures in orthodontic practice,^25^ facial landmark,^26^ removable orthodontic appliance,^28^ and cephalometric tracing.^38^ Four simulations, reported in six articles, were designed to facilitate orthodontic treatment procedures as a main purpose;^24^ ^,^ ^33^ ^-^ ^36^ ^,^ ^39^ however, they could be applied for training dental students or residents in orthodontic education. 

### EDUCATIONAL OUTCOMES OF TECHNOLOGY-ENHANCED SIMULATIONS INCLUDED IN THIS REVIEW

Two experimental studies demonstrated cognitive improvement of participants in orthodontic diagnostics and treatment planning after interacting with the simulations.^27^ ^,^ ^30^ In addition, orthodontic residents and orthodontists tended to have positive perceptions toward the use of simulations.^25^ ^,^ ^28^ ^,^ ^29^ ^,^ ^31^ They believed that they could gain confidence in orthodontic treatment procedures with the simulation.^25^ Participants were also likely to report high acceptance of the simulation in improving diagnostic competence.^29^ There were eight simulations, reported in ten publications, which were designed for the improvement of cognitive domain in orthodontic practice.^24^ ^,^ ^26^ ^,^ ^32^ ^-^ ^39^ However, no evidence of learning outcome evaluations has been provided in these articles. 

Three articles reported technology-enhanced simulations designed for the enhancement of psychomotor skills in orthodontic practice. One article reported the implementation of haptic technologies into an orthodontic simulation, in which users were allowed to perform the required steps of orthodontic bracket placement on a virtual patient.^32^ However, the article had not yet demonstrated the evidence of its educational impact on the improvement of learner competence. Two articles reported the application of AR for training orthodontic bracket placement.^30^ ^,^ ^37^ However, only one study performed a data collection process and presented the enhancement in the accuracy of bracket placement with the intervention, when compared with the conventional approach.^30^ These articles supported the implementation of simulations in improving psychomotor skills in orthodontic education. 

Overall, the included articles tended to support the use of technology-enhanced simulations in teaching and training orthodontic residents or orthodontists. When considering the 16 included publications according to the Kirkpatrick model,^40^ two experiments reported positive learning outcomes from the evaluation at a learning level.^27^ ^,^ ^30^ Four studies positively demonstrated the outcome evaluation focusing on a reaction level.^25^ ^,^ ^28^ ^,^ ^29^ ^,^ ^31^ Ten articles were published without reporting the outcome evaluation in terms of orthodontic education.^24^ ^,^ ^26^ ^,^ ^32^ ^-^ ^39^ None of the articles performed the evaluation of learning outcomes at behaviour and results levels.

## DISCUSSION

This scoping review was conducted to comprehensively analyze evidence of the use of technology-enhanced simulation-based learning in orthodontic practice. An increase in publications of technology-enhanced simulations in orthodontic education was found. In addition, the recent publications were more likely to report evidence supporting positive impact of technology-enhanced simulations as learning or training tools in orthodontic education. This rising trend was also found in the use of serious games in dental education,^4^ which could be resulting from the advancement of 3D modeling and computer graphic technologies. The findings retrieved from included articles demonstrated positive impact of technology-enhanced simulations in orthodontic education in terms of both cognitive and psychomotor skills. These results were consistent with the impact of digital simulations, including VR and AR, in other areas of dental education.^15^ ^,^ ^41^ Technology-enhanced simulations can be considered as very supportive in dentistry, including orthodontics, in which the integration of knowledge and hand skills is required for most of the dental treatments. Consequently, although limited, the existing evidence suggests the design and implementation of technology-enhanced simulations in orthodontic education. 

Several key strengths of technology-enhanced simulations should be considered. Firstly, they allow users to perform required tasks repetitively until the expected outcomes are achieved.^42^ ^,^ ^43^ In addition, with immediate feedback, those simulations can also support users to conduct self-directed learning, and therefore the needs of one-to-one support from dental instructors can be reduced.^44^ The concept of task repetition in improving learning competencies can be explained by the ‘role of failure’.^45^ Although this model is a game-based theory, it could well explain the learning process within the simulations. Students are required to rethink or reperform their tasks based on feedback received from the failure, which will lead to the improvement of knowledge and skills. Simulators and VR can simulate learning situations, where learners can improve their knowledge and skills in safe environment.^46^ ^,^ ^47^ This could reduce a risk of treatment, leading to the enhancement of patient safety in orthodontic practice. 

The COVID-19 pandemic promoted negative impact on orthodontic training. With a decrease in the number of dental patients and treatment visits due to either fear and anxiety of COVID19 infection or financial problems,^10^ ^,^ ^12^ orthodontic residents may not be able to gain sufficient experience of clinical training. Academic staff and educators are required to consider appropriate replacements for orthodontic training in clinical settings. In addition to serious gaming,^4^ ^,^ ^48^ technology-enhanced simulations should be considered to support residents in improving their orthodontic competence, as suggested according to the findings of this review. They could improve their knowledge and skills through repetitive tasks of learning activities within the simulations, which could prepare them for orthodontic training in clinical settings leading to the enhancement of patient safety. In addition, any orthodontic skills that may be insufficient from clinical training can be fulfilled with high-fidelity computer-based simulations.

When considering the Kirkpatrick model,^40^ the common types of the outcome evaluation of the articles included in this scoping review appeared to be similar to research in other areas of dental education, which were reaction and learning levels.^15^ ^,^ ^41^ There seemed to be no research evaluating the outcome at behaviour and results levels that could be considered as significant impact of the simulation development. In addition, while Bloom’s affective domain should be required for orthodontic practice, none of the included articles discussed the enhancement of this competence, although simulation-based pedagogical approaches can be considered as effective in improving these skills.^49^ Consequently, further design and development of high-fidelity computer-based simulations are necessary to simulate actual patient tasks, as well as research with robust design (e.g. well-blinded randomized controlled trials) to confirm these outcome evaluation in orthodontic education.

A few limitations were identified when conducting this scoping review. While the simulations designed for orthodontic treatment as a primary purpose could be adapted for training residents,^24^ ^,^ ^33^ ^-^ ^36^ ^,^ ^39^ the evidence of their outcome evaluation was not reported in these articles. Therefore, this scoping review cannot summarize the effectiveness of the implementation of orthodontic treatment simulations for teaching and training purposes. As this review had an emphasis on the outcome evaluation in orthodontic education, it was necessary to understand how to design training activities with simulations for training orthodontists or residents. For instance, they should be evaluated whether to be used as a bridge between classroom settings and clinical practice or a supplementary to both of them. Therefore, further original articles should be conducted to compare the effectiveness of the orthodontic treatment simulations adapted for training and the ones designed specifically for educational purposes, as well as to summarize how they should be implemented for the higher effectiveness in orthodontic practice. Furthermore, non-English search terms and other databases should be considered to further identify publications in other languages.

## CONCLUSION

Limited evidence identified in this scoping review has been generated to demonstrate the effectiveness of technology-enhanced simulations in orthodontic practice, although some studies reported no significant difference of the outcome evaluation, when comparing with traditional approaches. In addition, the outcome evaluations of technology-enhanced simulations in orthodontic practice had not yet been reported in a number of included articles. Consequently, further research should be required to confirm positive educational impact of technology-enhanced simulations on orthodontic education.
